# What is new in emergencies, trauma and shock? Studying stress in emergency medicine

**DOI:** 10.4103/0974-2700.55322

**Published:** 2009

**Authors:** Luciano Santana-Cabrera

**Affiliations:** Intensive Care Unit, Universitary Hospital Insular in Gran Canaria, Las Palmas de Gran Canaria, Canary Islands, Spain

The Emergency Departments (EDs) provide medical care to a population with increased needs for preventive care and limited access to screening and preventive interventions linked to stress. EDs are well positioned to provide data on several aspects of public health and give an opportunity to initiate preventive services for many people, to identify preventive interventions and, secondarily, to recommend areas in which research into the efficacy and cost-effectiveness of interventions are needed. The adoption of these initiatives may improve the health of this vulnerable population.

Patients are not always satisfied with the care received at EDs. More attention needs to be paid to the specific needs and expectations of the nonurgent group of patients who make up the majority of attenders at many EDs. Identifying areas for quality improvements are important to know where to take action. These findings may facilitate the work with changing attitudes and working routines, which are needed to deliver effective care and to improve the patients' perceptions of quality of care at EDs.

In 2000, the Society for Academic Emergency Medicine Public Health and Education Task Force developed recommendations for prevention, screening and counseling activities to be conducted in EDs.[1] In the ED setting, the evidence is sufficient to support offering preventive interventions, assuming sufficient resources are available, as the alcohol, human immunodeficiency virus and hypertension screening and smoking cessation counseling.[2]

There is a lack of information about the prevalence of stress, types of stressful situations and the relationship with other health issues in the ED population. This lack of knowledge of current evidence causes effective preventive and screening interventions from having not been widely adopted in the EDs.

For example, the unexplained chest pain is a common reason for emergency hospital admission and generates considerable health care costs for society. These patients report perceived stress at work and more prevalence of overweight.[3] In patients referred for chest pain or palpitations to an ED, the cardiac conditions are rare and the prevalence of panic and somatoform disorders is about three times higher than that of cardiac disease. This illustrates the importance of having a strategy to identify psychiatric disorders.[4]

The stress induces endocrine and immune changes as elevated levels of serum corticosterone and nerve growth factor and the chronic stressors suppress immune function and increase a host's susceptibility to disease. Also, the seasonal changes influence the immune function and the increased blood concentrations of adrenocortical steroids in response to them compromise immune function and accelerate catabolic mechanisms. Thus, there are mechanisms in some animals to combat seasonal stress-induced immunocompromise as a temporal adaptation to promote survival. In relation with this, the incidence and mortality of sepsis and severe sepsis are seasonal and consistently highest during the winter, predominantly related to respiratory sepsis.[5]

Further research should be directed not only to more efficient ways of identifying organic causes of disease but also to a more systematic evaluation of their symptoms, or the potential effects of lifestyle counseling in this large ED-patient population.

In this manner, Nirenberg *et al.*[6] studied the prevalence of stress, types of stressful situations and the relationship with other health issues within the ED population. Although they examined the stress in terms of only a brief self-reported measure, they found that almost half of the ED patients not only reported experiencing frequent stress but also reported many of the pshychosocial causes of stress (work, relationship and finances), health problems associated with stress (overweight and depression) and unhealthy coping strategies (cigarette and marijuana use) associated with experiencing persistent stress.

Brief interventions delivered to ED patients can be efficacious in reducing some unhealthy behaviors. There are articles that provide an overview of health promotion and disease and injury prevention concepts, discussing examples of innovative emergency medicine-based preventive activities, including prevention in the prehospital setting.[7]

It is necessary to integrate proven preventive and other public health initiative interventions into the ED setting in the emergency medicine core curriculum to configure clinical information systems to facilitate public health interventions and to use ancillary ED personnel to enhance delivery of public health interventions and obtain successful funding for these initiatives.

Smoking is the leading cause of preventable death and illness in developed countries and thus the Task Force considers it essential for emergency physicians to provide some recommendations for tobacco control practice.[8] There is strong evidence, in the primary care setting, that smoking cessation screening and counseling are effective, but limited data exist for ED-based practice. But, based on the relative ease of intervention and likely efficacy, routine screening of all patients for tobacco use and referral of smokers to primary care and cessation programs are recommended.[9]

There are authors who recommend the screening and brief intervention for alcohol-related problems in the ED setting because preventing the mortality and morbidity secondary to alcohol-related illnesses/injuries decreases consumption, diminishes the visits of the patients to ED and hospitalizations, decreases the social consequences and increases referrals for follow-up and/or treatment.[10]

Besides the stress originated by the physical pain or the incapacity to be able to move or to communicate with the outside, we also have inadequate information that is offered to the patients and the little preoccupation by the professional about the psychological aspects of them. For this reason, it is important to be interested in aspects like the problems or feelings of the patients, reinforcing the information on the procedures to perform, asking for their previous consent and to give them the name of the doctor and the nurse who are treating them. All this would increase the quality perceived by the patient.[11] Also, it is important to know the family needs of patients admitted to EDs. The ED is one of the places in a hospital where family members suffer the most. It is probably that in the majority of the cases family members visiting the patient in the ED fail to understand what doctors say about the prognosis, diagnosis and treatment of the patient they are taking care of, causing a high prevalence of emotional disorders in the family members.

It is not necessary to forget the stress in the personnel who work in EDs, because a relationship exists between working conditions and stress, anxiety, depression and quality of life. The stress at work is connected with the intensity of the burn out and it is a common problem among people who work in an ED. The presence of risk factors derived from work organisation within the work place increases the probability of presenting the burnout syndrome and, above all, the emotional exhaustion.[12] The most stressful aspects of work are dealing with management, insufficient staffing, workload pressures and staff supervision. The number of foreigners using EDs has risen in recent years, looking for nonurgent services, as this is the only facility to which they are entitled to refer for medical treatment. This fact underlines the need to reform healthcare legislation in such a way as to entitle every foreigner to be treated by a Family Physician. This would reduce both waiting times in the EDs and the irritation of medical personnel who are called upon to deal with nonurgent cases. This fact can cause medical errors where decisions are made under pressure and with incomplete information, with a potential risk of misidentification of patients in the ED. Factors such as the number of patients, the urgency of individual cases, the ability of patients to communicate, language barriers, low staff to patient ratios and time pressures can all contribute to the risk. Errors often occur, not as result of failure of a single entity within the system but as a result of a break down of the system. Whereas in some cases technological situations can improve the situation, in others, they could lead to increased errors.[13]

Emergency personnel are continuously under stress because of overcrowded departments, severity of cases and their work schedules. In addition to this work stress, irregular social and family life is the main component of the ongoing burn out process in these professionals. Burn out is the end point of the process, which is complicated by the loss of professional enthusiasm and satisfaction and a negative behavioral approach to the patients.[14] The clinical work impacts most upon family life, social life and emotional health, and it is associated with anxiety, depression and stress, which cause maladaptive strategies (alcohol, drugs, disengagement) by the professional. Also, the malpractice claims consume an enormous amount of financial resources within the medical system, generating billions of dollars in defensive medical costs. The frequent and well-publicised litigation causes a certain nervousness among emergency physicians, which impinges on their attitude to work and their clinical practice. Resource factors that have the greatest impact on job satisfaction include availability of emergency room physicians, access to hospital technology and emergency beds and stability of financial resources.[15] Fellows are significantly reducing their clinical workload largely in response to excessive workload and lack of resources. These findings have important implications for professional longevity and work force planning.[16]
